# Supplementary material for: A community audit of error control in crosslinking mass spectrometry
Source: bioRxiv. 2026 Oct 28:2025.04.27.649519. Preprint. [Version 2] doi: 10.1101/2025.04.27.649519 (PMC13733680; doi:10.1101/2025.04.27.649519)
Supplement: Supplementary file 1 [file NIHPP2025.04.27.649519v2-supplement-1.pdf]

bioRxiv preprint doi: https://doi.org/10.1101/2025.04.27.649519; this version posted October 28, 2026. The copyright holder for this preprint (which was not certified by peer review) is the author/funder, who has granted bioRxiv a license to display the preprint in perpetuity. It is made available under a CC-BY 4.0 International license.

indicates engines without a computable decoy FDR at CSM level. **c,** Observable false-target rate at CSM (circle) and PPI (triangle) level for each engine. Blue: *E. coli–H. sapiens*; orange: *E. coli–M. pneumoniae*. Engines with a large CSM-to-PPI increase export disproportionate numbers of false PPIs. Dashed line marks 2%. **d,** CSM- and PPI-level observable false-target rates for XlinkX PD2.5 and XlinkX PD3.3 (*E. coli–H. sapiens*). The substantial reduction between versions demonstrates the impact of community driven benchmarks. Dashed line marks 2%

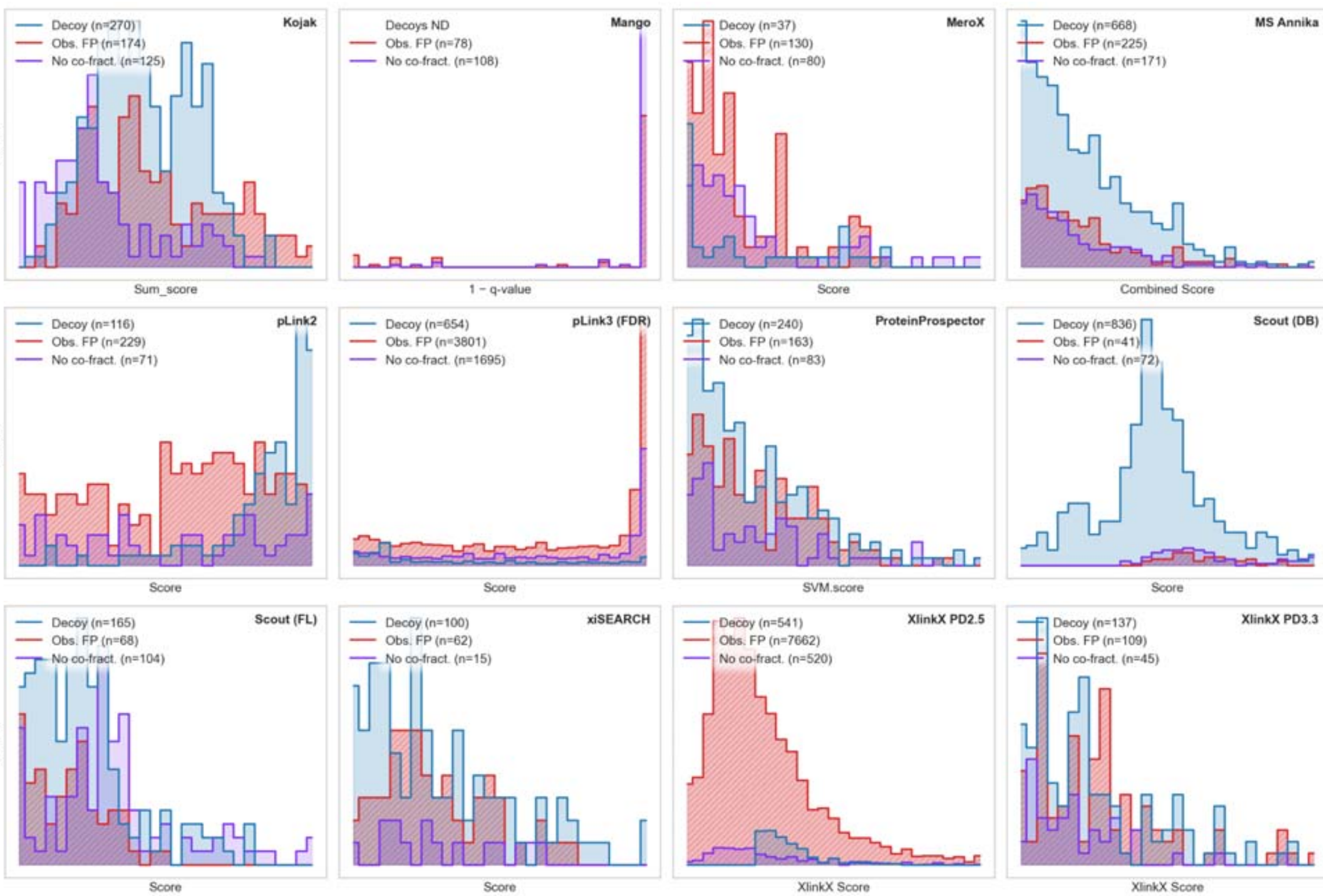

Figure S1 Score Distributions of Observable False Positives and Decoys in *E. coli–H. sapiens* dataset

One panel per pipeline, showing the score distribution of decoy matches (blue), of TT matches to a human entrapment protein (red) and of TT *E. coli–E. coli* pairs whose proteins never co-fractionate (purple). The three classes are disjoint. If decoys model false target matches correctly, the blue and red distributions should have the same shape and the blue count should be about twice the red. Pipelines where the red distribution instead extends to high scores, or outnumbers the decoys, have a decoy population that no longer represents their error.

bioRxiv preprint doi: https://doi.org/10.1101/2025.04.27.649519; this version posted October 28, 2026. The copyright holder for this preprint (which was not certified by peer review) is the author/funder, who has granted bioRxiv a license to display the preprint in perpetuity. It is made available under a CC-BY 4.0 International license.

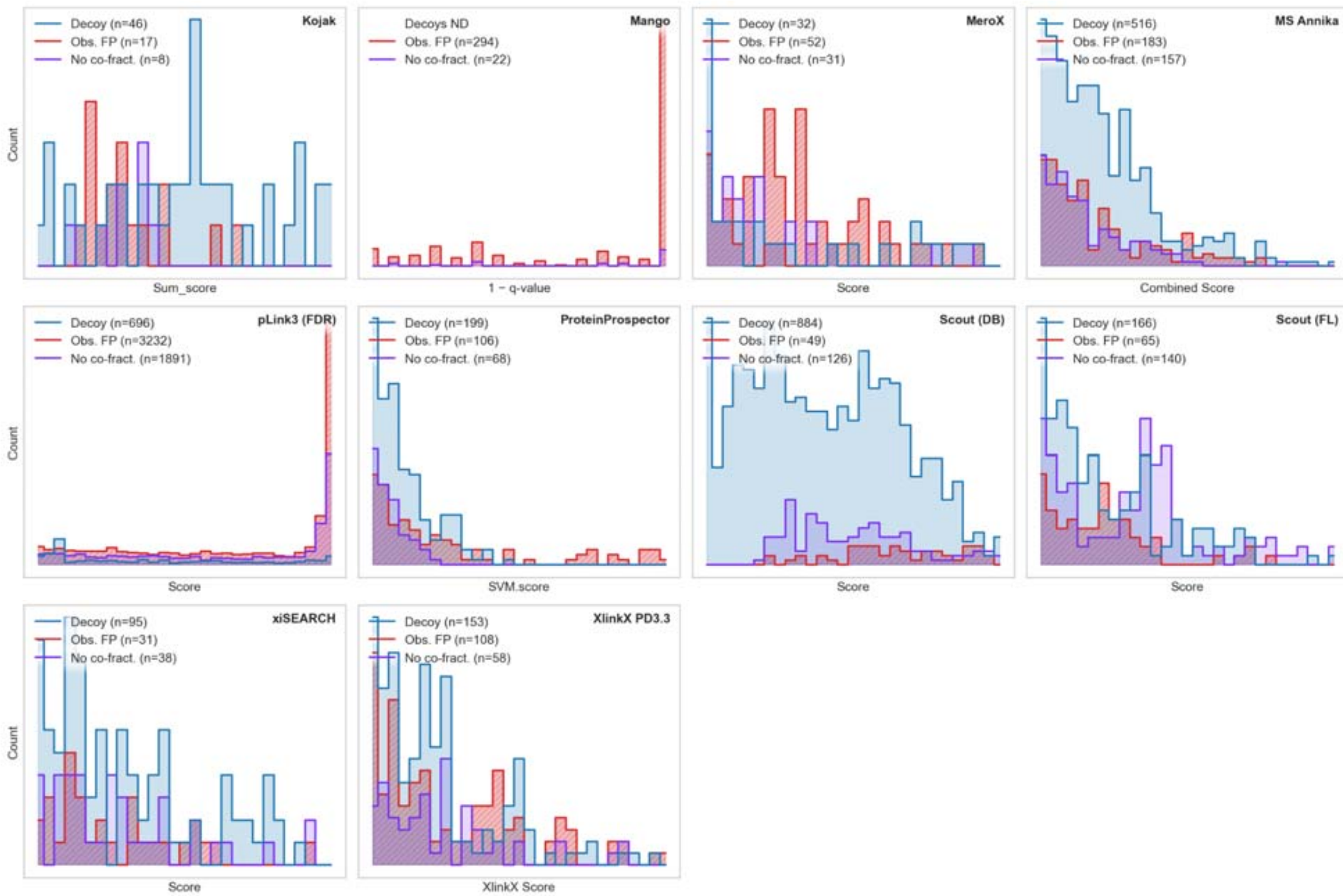

Figure S2 Decoy vs known false-positive score distributions (*E. coli*–*M. pneumoniae*, CSM)

The *E. coli*–*M. pneumoniae* counterpart of Figure S1, with the ground truth supplied by the other reference dataset: a CSM whose spectrum came from one organism but was matched to the other (red). Decoy matches are blue; *E. coli*–*E. coli* pairs that never co-fractionate are purple. Reading the two figures side by side separates pipeline behaviour from any quirk of a single ground-truth construction. Note that the expected decoy-to-false-positive ratio is not 2 here: only the cross-species subset of false positives is observable, and the two organisms contribute very unequal shares of the database.

bioRxiv preprint doi: https://doi.org/10.1101/2025.04.27.649519; this version posted October 28, 2026. The copyright holder for this preprint (which was not certified by peer review) is the author/funder, who has granted bioRxiv a license to display the preprint in perpetuity. It is made available under a CC-BY 4.0 International license.

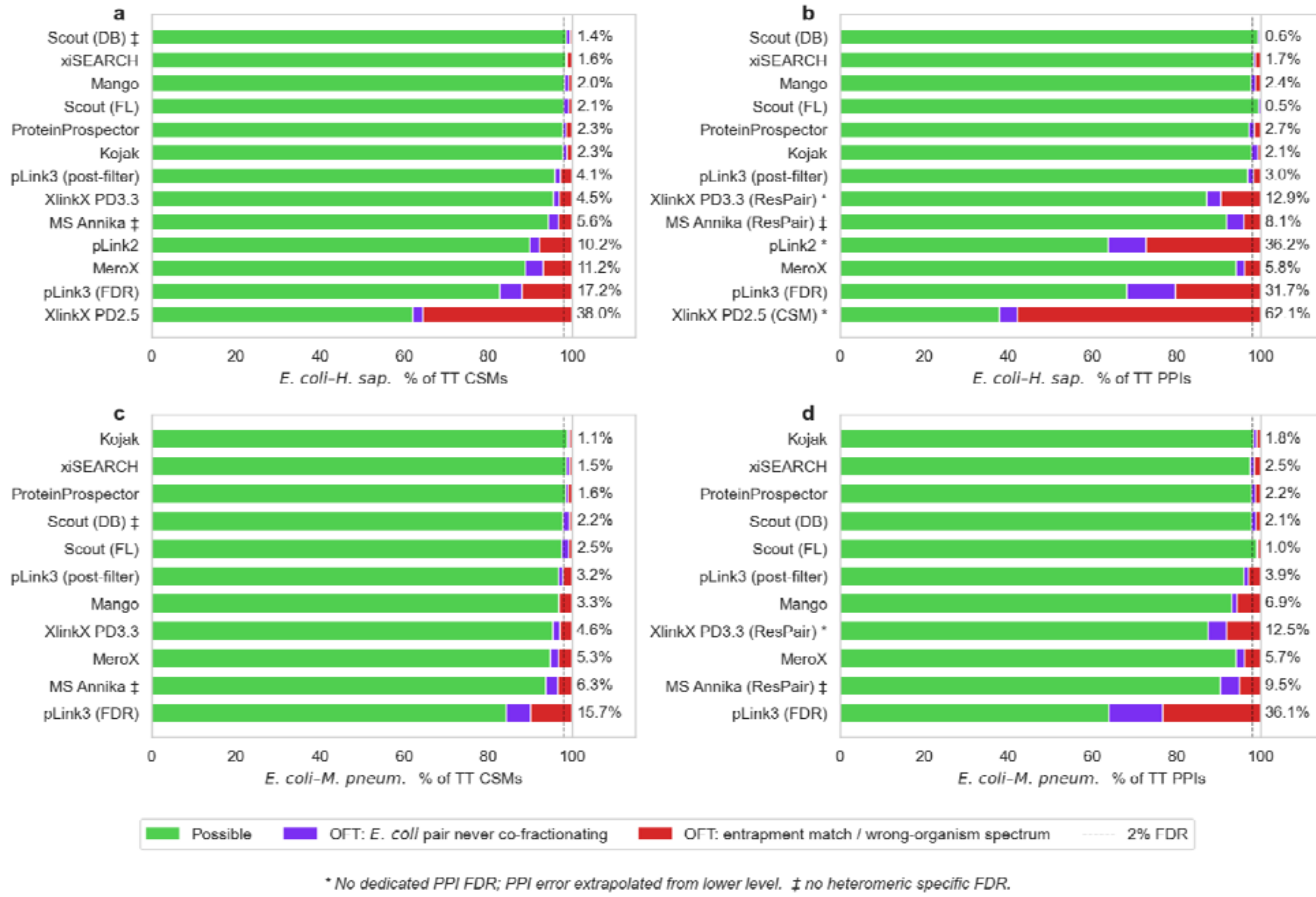

Figure S3 Observable false target (OFT) breakdown

Composition of the accepted target–target matches for each pipeline, as a percentage of TT. **(a, b)** *E. coli–H. sapiens* at CSM and PPI level. **(c, d)** *E. coli–M. pneumoniae* at CSM and PPI level. Bars run possible (green), then *E. coli* pairs that never co-fractionate (purple), then entrapment matches or wrong-organism spectra (red). The number at the right of each bar is the total OFTR. Each dataset is ranked on its own CSM total, and its PPI panel repeats that order so a pipeline sits at the same height in both. Pipelines marked * have no dedicated PPI FDR, with PPI error extrapolated from a lower level; ‡ marks pipelines that do not split FDR by link type.

bioRxiv preprint doi: https://doi.org/10.1101/2025.04.27.649519; this version posted October 28, 2026. The copyright holder for this preprint (which was not certified by peer review) is the author/funder, who has granted bioRxiv a license to display the preprint in perpetuity. It is made available under a CC-BY 4.0 International license.

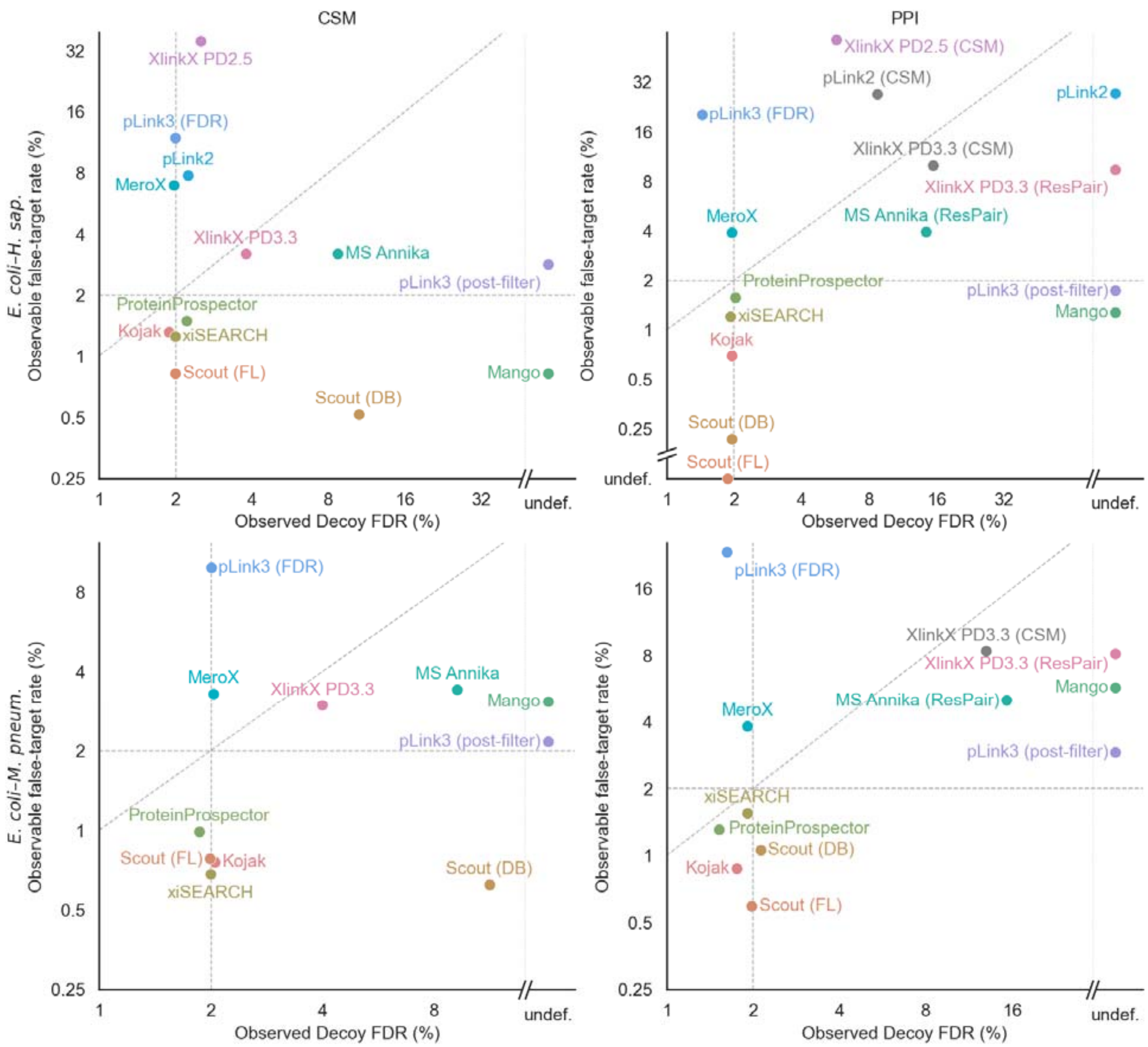

Figure S4 Observable error vs observed decoy FDR

Each point is one search pipeline at its declared 2 % FDR; x is the observed decoy FDR, y the OFTR. Rows are the two datasets (*E. coli–H. sapiens* top, *E. coli–M. pneumoniae* bottom), columns the level (CSM left, PPI right). Points above the diagonal under-report their error. Mango, which does not use decoys for error estimate, and PPI level information without supplied decoys are placed in a separate column at the right. Scout (FL) *E. coli–H. sapiens* did not produce any detectable false positives and was put on the x-axis. These are marked as undefined.

bioRxiv preprint doi: https://doi.org/10.1101/2025.04.27.649519; this version posted October 28, 2026. The copyright holder for this preprint (which was not certified by peer review) is the author/funder, who has granted bioRxiv a license to display the preprint in perpetuity. It is made available under a CC-BY 4.0 International license.

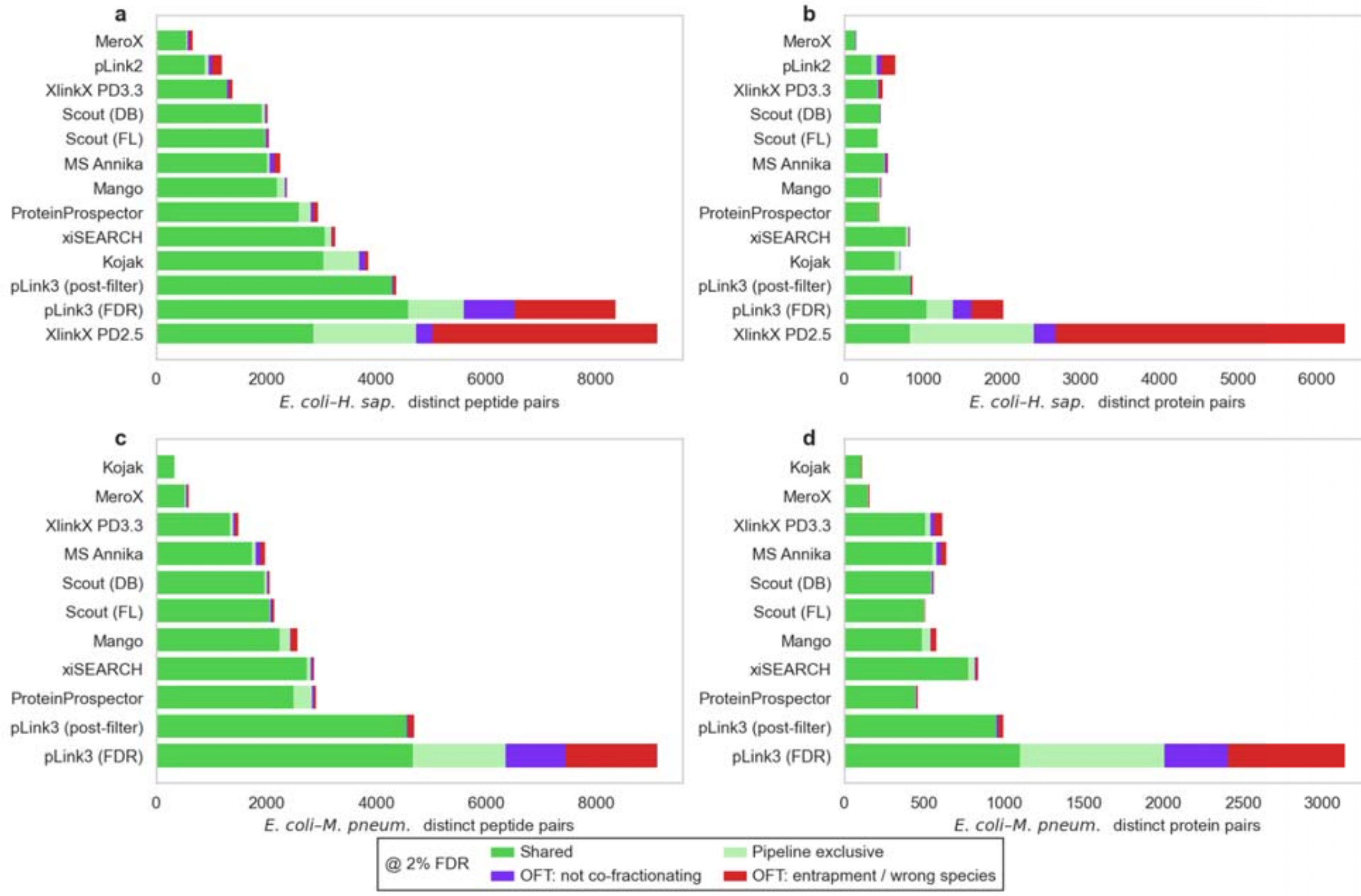

Figure S5 Cross-pipeline overlap

Overlap of reported pairs across pipelines, for both datasets and both levels.
Rows are the datasets (*E. coli–H. sapiens* top, *E. coli–M. pneumoniae* bottom); columns are the level (unique peptide pairs left, unique protein pairs right). Bars split into shared vs unique and possible vs observable false positives (FP). Observable FP are again split into either an entrapment or cross-species match, or an *E. coli* pair whose two proteins never co-fractionated. Each row shares one engine order, taken from its peptide-pair panel, so a pipeline sits at the same height in both of its panels.

bioRxiv preprint doi: https://doi.org/10.1101/2025.04.27.649519; this version posted October 28, 2026. The copyright holder for this preprint (which was not certified by peer review) is the author/funder, who has granted bioRxiv a license to display the preprint in perpetuity. It is made available under a CC-BY 4.0 International license.

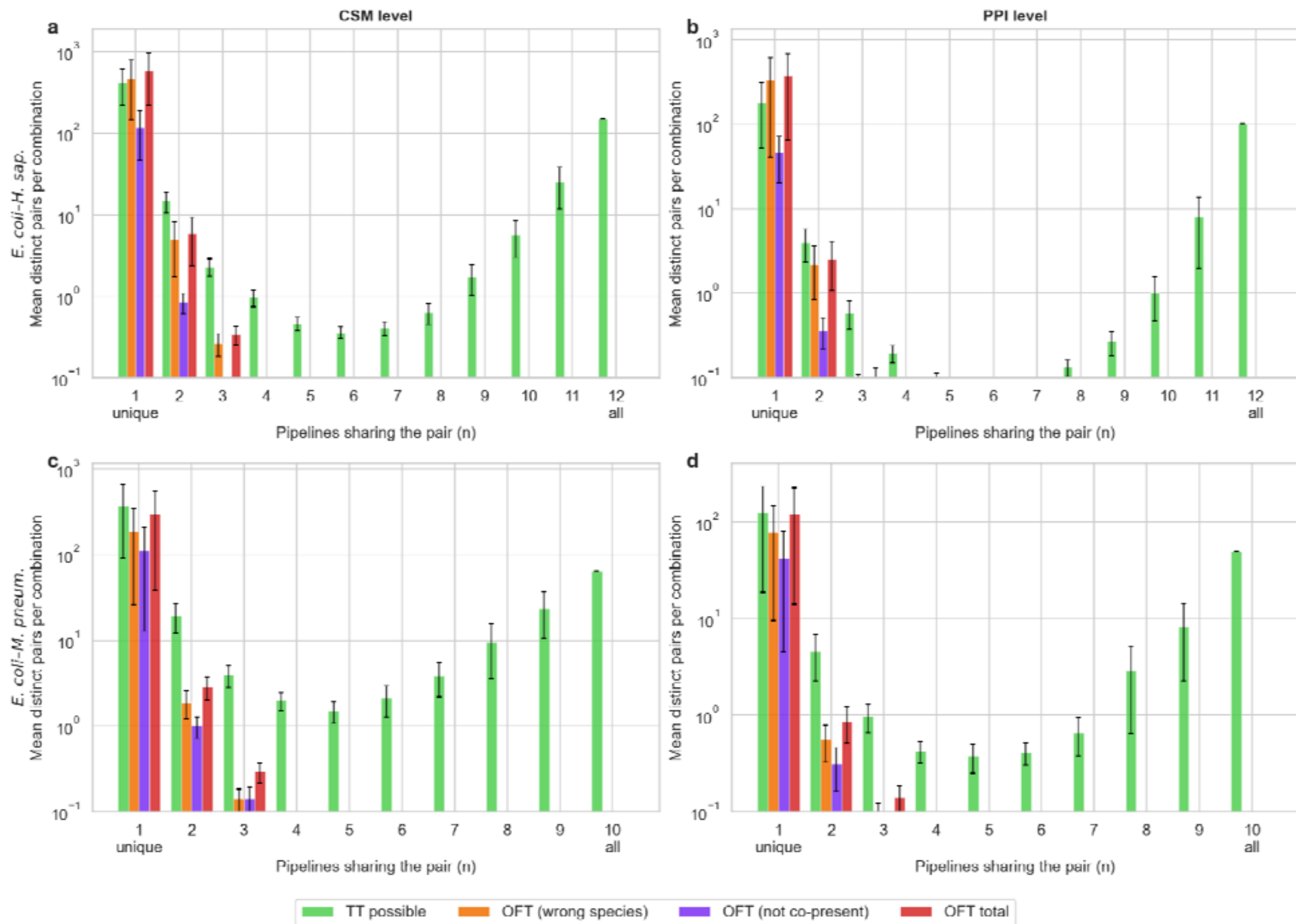

Figure S6 Cross pipeline overlap by pipeline count

Extension of Figure S5 (cross-pipeline overlap): instead of one shared/unique split per pipeline, pairs are grouped by how many of the N pipelines report them (pLink3 post-filter excluded; N = 12 for E. coli–H. sapiens, N = 10 for E. coli–M. pneumoniae). Rows are the datasets (E. coli–H. sapiens top, E. coli–M. pneumoniae bottom); columns are the level (CSM left, PPI right) — the same layout as Figure S5. (a) E. coli–H. sapiens, CSM level. (b) E. coli–H. sapiens, PPI level. © E. coli–M. pneumoniae, CSM level. (d) E. coli–M. pneumoniae, PPI level.

For each sharing level n = 1 (unique to one pipeline) through n = N (reported by every pipeline), bars give the mean distinct-pair count across all C(N, n) specific n-pipeline combinations — not just the combinations that happen to occur — split into TT possible (green), observable false target (OFT) from a wrong-species/entrapment match (orange), OFT from an E. coli pair that never co-fractionates (purple), and the two OFT reasons pooled (OFT total, red). Error bars are the standard error across combinations; they are absent at n = N, where only one such combination exists. The y-axis is log-scaled, so a category with zero pairs at a given n is simply not drawn.

bioRxiv preprint doi: https://doi.org/10.1101/2025.04.27.649519; this version posted October 28, 2026. The copyright holder for this preprint (which was not certified by peer review) is the author/funder, who has granted bioRxiv a license to display the preprint in perpetuity. It is made available under a CC-BY 4.0 International license.

Supplemental Table S1 Single entrapment CSM level data per search engine

Search Pipeline	TT	TD	DD	Decoy FDR	OFT Species	OFTR Species	OFT not copresent	OFTR combined
Kojak	13204	259	11	1.88%	174	1.32%	125	2.26%
Scout (FL)	8268	165	0	2.00%	68	0.82%	104	2.08%
Scout (DB)	7919	834	2	10.51%	41	0.52%	72	1.43%
xiSEARCH	4931	99	1	1.99%	62	1.26%	15	1.56%
Protein-Prospector	10869	240	0	2.21%	163	1.50%	83	2.26%
Mango	9497	0	0	0.00%	78	0.82%	108	1.96%
MS Annika	7034	640	28	8.70%	225	3.20%	171	5.63%
MeroX	1877	37	0	1.97%	130	6.93%	80	11.19%
pLink2	2946	91	25	2.24%	229	7.77%	71	10.18%
pLink3 (FDR)	31941	646	8	2.00%	3801	11.90%	1695	17.21%
pLink3 (post-filter)	25163	0	0	0.00%	714	2.84%	323	4.12%
XlinkX PD2.5	21543	12	529	2.51%	7662	35.57%	520	37.98%
XlinkX PD3.3	3416	133	4	3.78%	109	3.19%	45	4.51%

Supplemental Table S2 Single entrapment PPI level data per search engine

Search Pipeline	TT	TD	DD	Decoy FDR	OFT Species	OFTR Species	OFT not copresent	OFTR combined
Kojak	719	14	0	1.95%	5	0.70%	10	2.09%
Scout (FL)	427	8	0	1.87%	0	0.00%	2	0.47%
Scout (DB)	463	9	0	1.94%	1	0.22%	2	0.65%
xiSEARCH	831	16	0	1.93%	10	1.20%	4	1.68%

bioRxiv preprint doi: https://doi.org/10.1101/2025.04.27.649519; this version posted October 28, 2026. The copyright holder for this preprint (which was not certified by peer review) is the author/funder, who has granted bioRxiv a license to display the preprint in perpetuity. It is made available under a CC-BY 4.0 International license.

Protein-Prospector	445	9	0	2.02%	7	1.57%	5	2.70%
Mango	467	0	0	0.00%	6	1.28%	5	2.36%
MS Annika	557	83	3	14.36%	22	3.95%	23	8.08%
MeroX	154	3	0	1.95%	6	3.90%	3	5.84%
pLink2	647	0	0	0.00%	176	27.20%	58	36.17%
pLink3 (FDR)	2019	29	0	1.44%	408	20.21%	233	31.75%
pLink3 (post-filter)	868	0	0	0.00%	15	1.73%	11	3.00%
XlinkX PD2.5	6354	8	355	5.71%	3674	57.82%	272	62.10%
XlinkX PD3.3	490	0	0	0.00%	46	9.39%	17	12.86%

Supplemental Table S3 Dual entrapment CSM level data per search engine

Search Pipeline	TT	TD	DD	Decoy FDR	OFT Species	OFTR Species	OFT not copresent	OFTR combined
Kojak	2247	46	0	2.05%	17	0.76%	8	1.11%
Scout (FL)	8343	166	0	1.99%	65	0.78%	140	2.46%
Scout (DB)	7858	883	1	11.22%	49	0.62%	126	2.23%
xiSEARCH	4555	93	2	2.00%	31	0.68%	38	1.51%
Protein-Prospector	10721	199	0	1.86%	106	0.99%	68	1.62%
Mango	9598	0	0	0.00%	294	3.06%	22	3.29%

bioRxiv preprint doi: https://doi.org/10.1101/2025.04.27.649519; this version posted October 28, 2026. The copyright holder for this preprint (which was not certified by peer review) is the author/funder, who has granted bioRxiv a license to display the preprint in perpetuity. It is made available under a CC-BY 4.0 International license.

MS Annika	5366	505	11	9.21%	183	3.41%	157	6.34%
MeroX	1580	32	0	2.03%	52	3.29%	31	5.25%
pLink3 (FDR)	32622	674	22	2.00%	3232	9.91%	1891	15.70%
pLink3 (post-filter)	25052	0	0	0.00%	541	2.16%	268	3.23%
XlinkX PD3.3	3637	149	4	3.99%	108	2.97%	58	4.56%

Supplemental Table S4 Dual entrapment PPI level data per search engine

Search Pipeline	TT	TD	DD	Decoy FDR	OFT Species	OFTR Species	OFT not copresent	OFTR combined
Kojak	114	2	0	1.75%	1	0.88%	1	1.75%
Scout (FL)	506	10	0	1.98%	3	0.59%	2	0.99%
Scout (DB)	565	12	0	2.12%	6	1.06%	6	2.12%
xiSEARCH	838	16	0	1.91%	13	1.55%	8	2.51%
Protein-Prospector	459	7	0	1.53%	6	1.31%	4	2.18%
Mango	578	0	0	0.00%	33	5.71%	7	6.92%
MS Annika	640	101	3	15.31%	32	5.00%	29	9.53%
MeroX	157	3	0	1.91%	6	3.82%	3	5.73%
pLink3 (FDR)	3140	53	2	1.62%	733	23.34%	402	36.15%
pLink3 (post-filter)	996	0	0	0.00%	29	2.91%	10	3.92%

bioRxiv preprint doi: https://doi.org/10.1101/2025.04.27.649519; this version posted October 28, 2026. The copyright holder for this preprint (which was not certified by peer review) is the author/funder, who has granted bioRxiv a license to display the preprint in perpetuity. It is made available under a CC-BY 4.0 International license.

XlinkX PD3.3	616	0	0	0.00%	50	8.12%	27	12.50%

Supplemental Table S5 Search pipeline configurations. Crosslinker, protease, fixed/variable modifications, precursor/fragment tolerance (4 ppm / 8 ppm), and requested FDR (2%) were identical across all twelve pipelines and are omitted from the table; see above for the deviations summarised here.

Pipeline	Version	Missed cleavages	Crosslinker-reactive residues	Peptide length	Decoy generation	Notes
Kojak	v2.0.2	2 (default)	default	default (~5–60)	default (reverse)	PPI-level scoring via iProphet (TPP v7.1.0 Albedo)
Scout (FL)	v2.0.0	2 (default)	K, protein N-term	min. 7	default (reverse)	
Scout (DB)	v2.1.0 †	3	K, S, T, Y (equal weight)	default (~5–60)	default (reverse)	
xiSEARCH	v1.8.13	2 (default)	K, protein N-term, + S/T/Y (reduced weight)	default (~5–60)	default (reverse)	
ProteinProspector	v6.5.2	2 (default)	K, protein N-term	default (~5–60)	randomisation	Rescoring/FDR via pre-release Touchstone (minPepLen=5, minProdIon.1=minProdIon.2=4, decoyScalingFactor=1)
MS Annika	v3.0.7 (PD v3.1.0.6	2 (default)	K, protein N-term	default (~5–60)	default (reverse)	

bioRxiv preprint doi: https://doi.org/10.1101/2025.04.27.649519; this version posted October 28, 2026. The copyright holder for this preprint (which was not certified by peer review) is the author/funder, who has granted bioRxiv a license to display the preprint in perpetuity. It is made available under a CC-BY 4.0 International license.

	38)					
MeroX	v2.0.2.5	2 (default)	default	7–23	default (reverse)	
Mango	v20260105	2 (default)	default	default (~5–60)	— (decoy-free)	
pLink2	v2.3.10	2 (default)	default	default (~5–60)	default (reverse)	Run in-house
pLink3	v3.0.17	2 (default)	default	default (~5–60)	default (reverse)	Third-party submitted results
XlinkX PD2.5	Proteome Discoverer 2.5	2 (default)	K, protein N-term	default (~5–60)	default (reverse)	Upstream Sequest HT search (20 ppm / 0.5 Da) + confidence filter precedes crosslink search
XlinkX PD3.3	PD 3.3.0.386	2 (default)	K, protein N-term	default (~5–60)	default (reverse)	Upstream Sequest HT search (10 ppm / 0.02 Da) + confidence filter precedes crosslink search
